# Cap-assisted snare capture technique for resection of fibrotic large polyps

**DOI:** 10.1016/j.vgie.2024.05.013

**Published:** 2024-06-05

**Authors:** Muhammad Nadeem Yousaf, Ibukunoluwa Oshobu, Syed F. Jafri, Fadi Bdair, Ahmed Saeed

**Affiliations:** 1 Division of Gastroenterology and Hepatology, Department of Medicine, University of Missouri–Columbia, Missouri, USA; 2 HCA Midwest Health, Kansas City, Kansas, USA

## Background

EMR of nonlifting or fibrotic polyps can be challenging. The snare resection of flat or depressed fibrotic areas is difficult because of failure of the snare to capture. We describe a technique for capturing fibrotic tissue using the distal attachment clear cap as an adjunct technique to EMR of nonlifting or fibrotic polyps. This is a similar concept to cap EMR, which requires a special kit. This technique uses the distal attachment clear cap that is commonly used in most EMR procedures.

## Endoscopic technique

The equipment needed for “cap-assisted snare capture” technique is a distal attachment clear cap and a soft braided hot snare. We recommend using a 10-mm soft braided hot snare as it allows better tissue capturing and easy maneuverability and minimizes the risks of perforation. In this case series, we described the resection of fibrotic tissue after EMR of large colon polyps (Video 1, available online at www.videogie.org). Before EMR referral, biopsy was not performed, nor was any attempt made to resect the lesions in the presented cases.

### Steps of the “cap-assisted snare capture” technique

Step 1: Apply the distal attachment cap at the tip of the endoscope.

Step 2: When a difficult-to-capture area is encountered during EMR, place a 10- to 15-mm snare around the fibrotic tissue.

Step 3: Maneuver the scope to position the cap on top of the open snare and apply suction.

Step 4: Simultaneously close the snare to capture the fibrotic tissue while continuing to apply suction.

Step 5: Release the suction and confirm the snare capture of the targeted tissue before resection.

**Figure 1:**
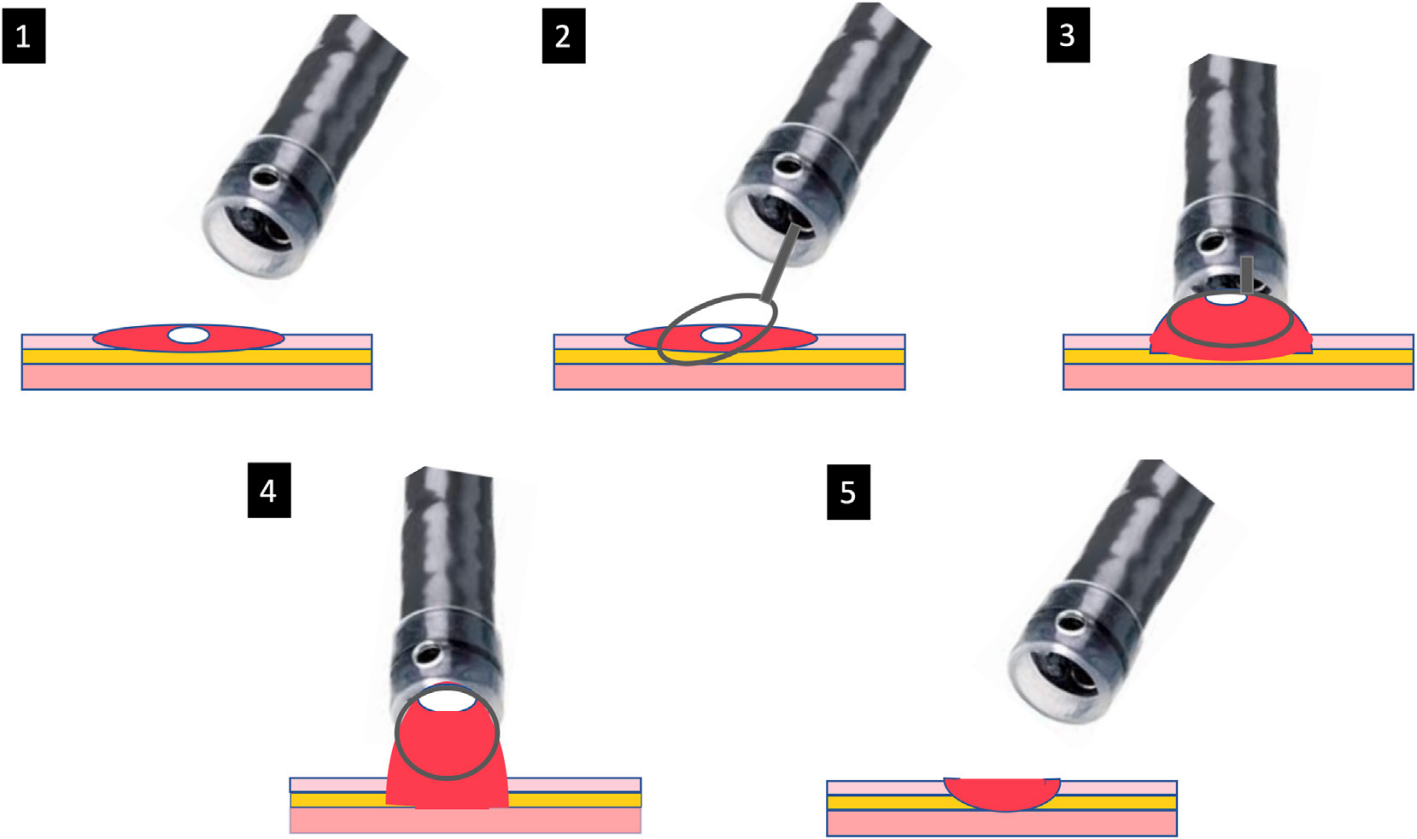
Illustration of the cap-assisted snare capture technique. Endoscopic evaluation of residual adenomatous tissue in the middle of a resected polyp after EMR (*panel 1*); a soft snare placement around the residual tissue (*panel 2*); endoscope maneuvering to position the cap on top of the open snare (*panel 3*); suction application with simultaneous snare closure to capture targeted tissue before resection (*panel 4*); and inspection of resection site for residual tissue, bleeding, and perforation (*panel 5*).

For large fibrotic areas, repeat steps 2 to 5 until all fibrotic tissue is resected (Fig. 1).

## Case 1

**Figure 2:**
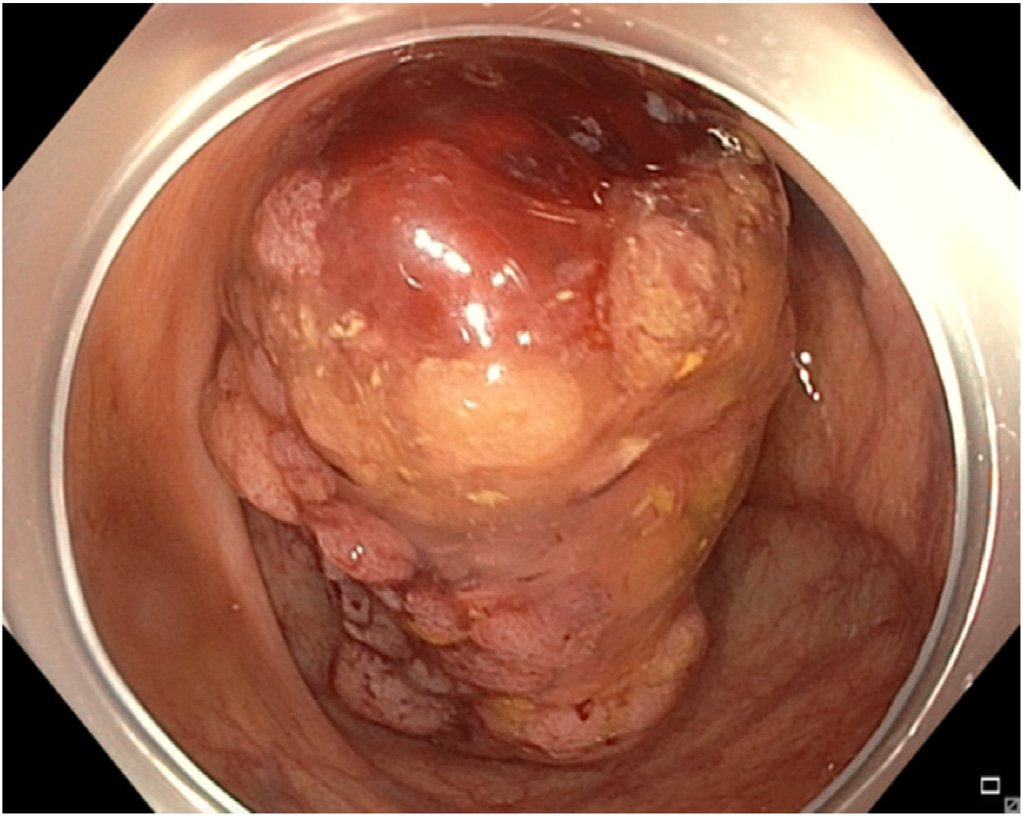
A 5-cm Paris Is polyp at the base of the cecum covered with mucus.

**Figure 3:**
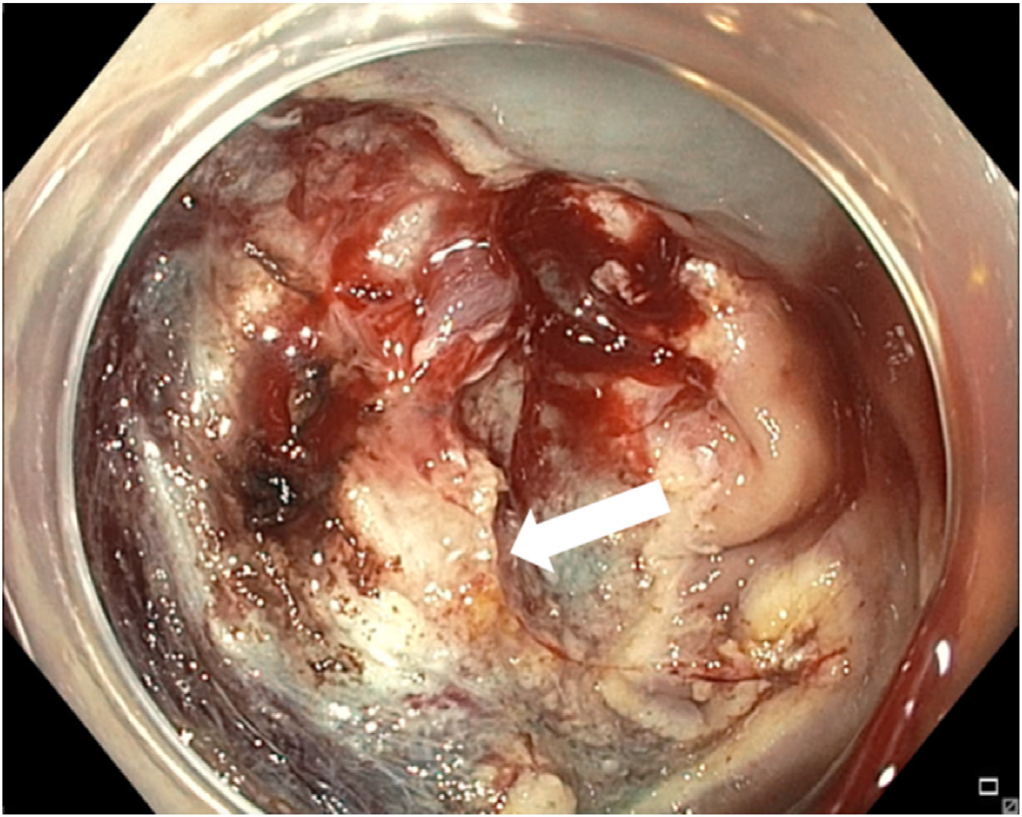
Residual scarred fibrotic tissue at the base of the polyp (*arrow*).

**Figure 4:**
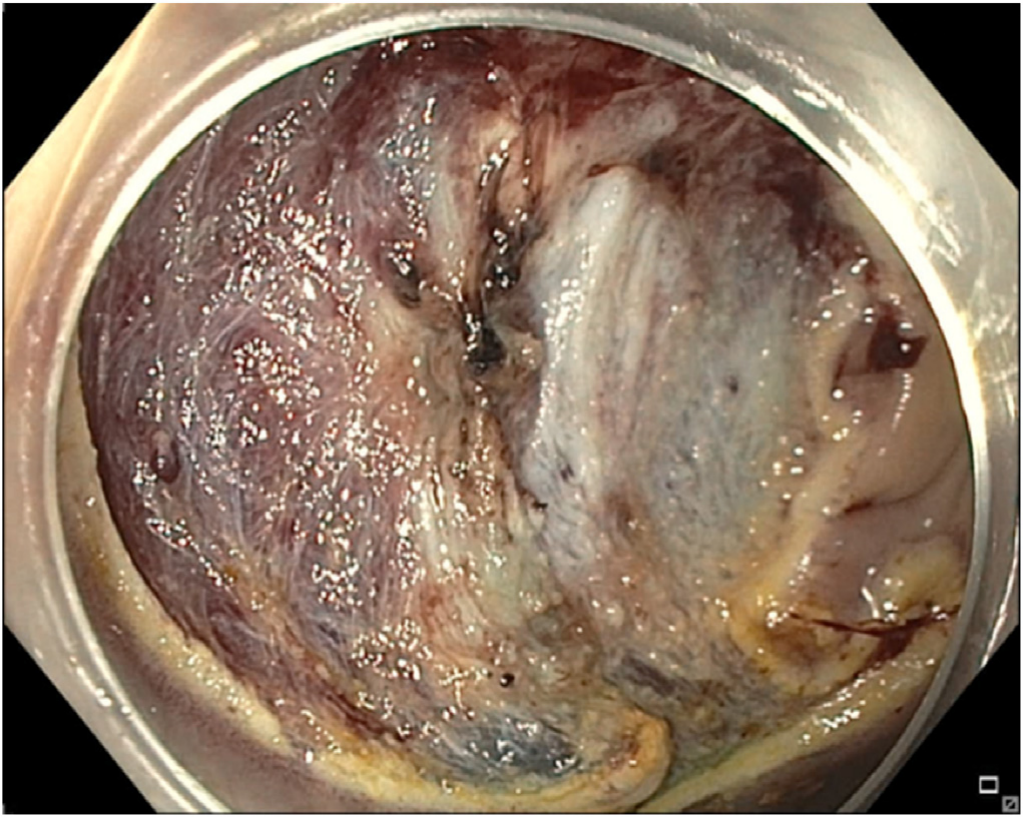
The base of the polyp after resection of fibrotic tissue using the cap-assisted snare capture technique.

A 65-year-old man was referred to our clinic for EMR of a 5-cm polyp at the base of the cecum. The polyp was classified as Paris Is and Japan NBI Expert Team (JNET) IIb (Fig. 2). It was covered with mucus. The polyp was raised with submucosal injection of conventional starch-based lifting solution, and piecemeal resection was performed using a 30-mm braided snare. Although the polyp size was 5 cm, its base was approximately 2 cm. Because of the semipedunculated nature of the polyp, a 30-mm snare was maneuvered around the polyp to perform EMR. There was a leftover scarred fibrotic tissue at the base of the polyp (Fig. 3). Multiple attempts to capture fibrotic tissue with the snare were unsuccessful because of sliding of the snare over fibrotic tissue. The cap-assisted snare capture technique was used for resection of residual fibrotic tissue. A 10-mm soft braided hot snare was placed around the fibrotic tissue, and it was suctioned into the cap while simultaneously closing the snare to capture the residual tissue. After confirming the snare capture of targeted tissue, it was resected using conventional hot EMR settings. No visible residual adenoma tissue was seen on careful examination of the resection site (Fig. 4). Snare tip soft coagulation of the normal resection margins was performed to decrease the risk of recurrence. The defect was closed with multiple through-the-endoscope clips.

## Case 2

**Figure 5:**
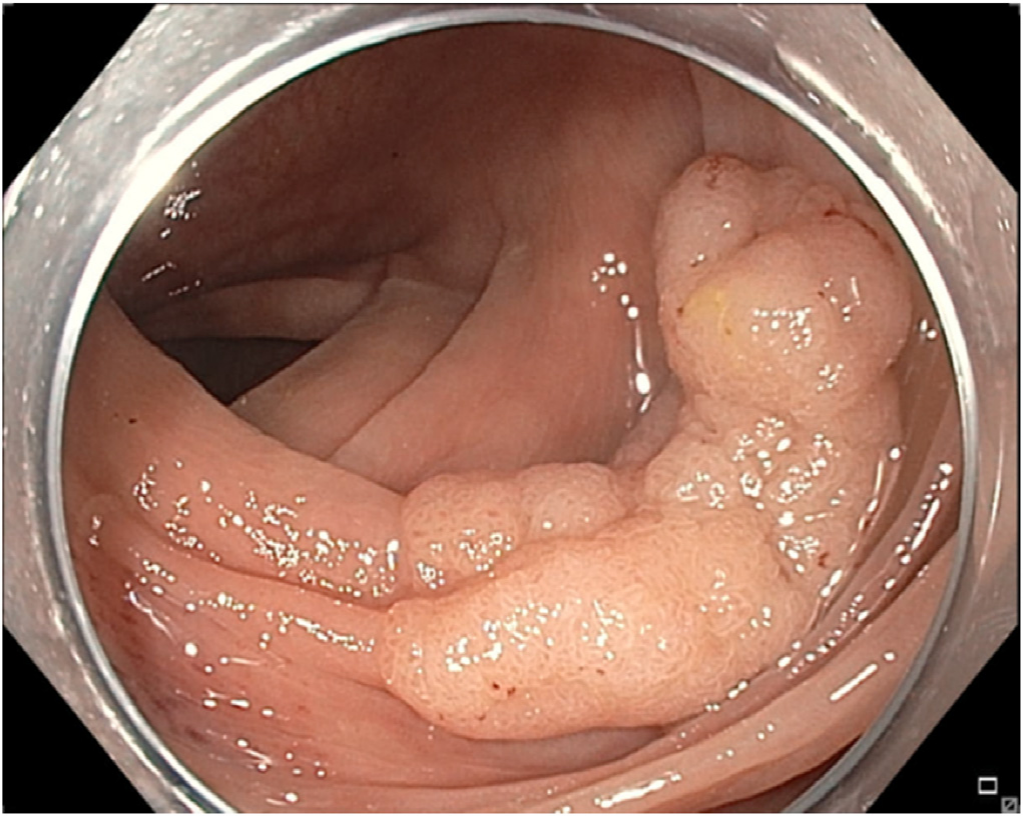
A 3-cm granular, laterally spreading tumor in the transverse colon.

**Figure 6:**
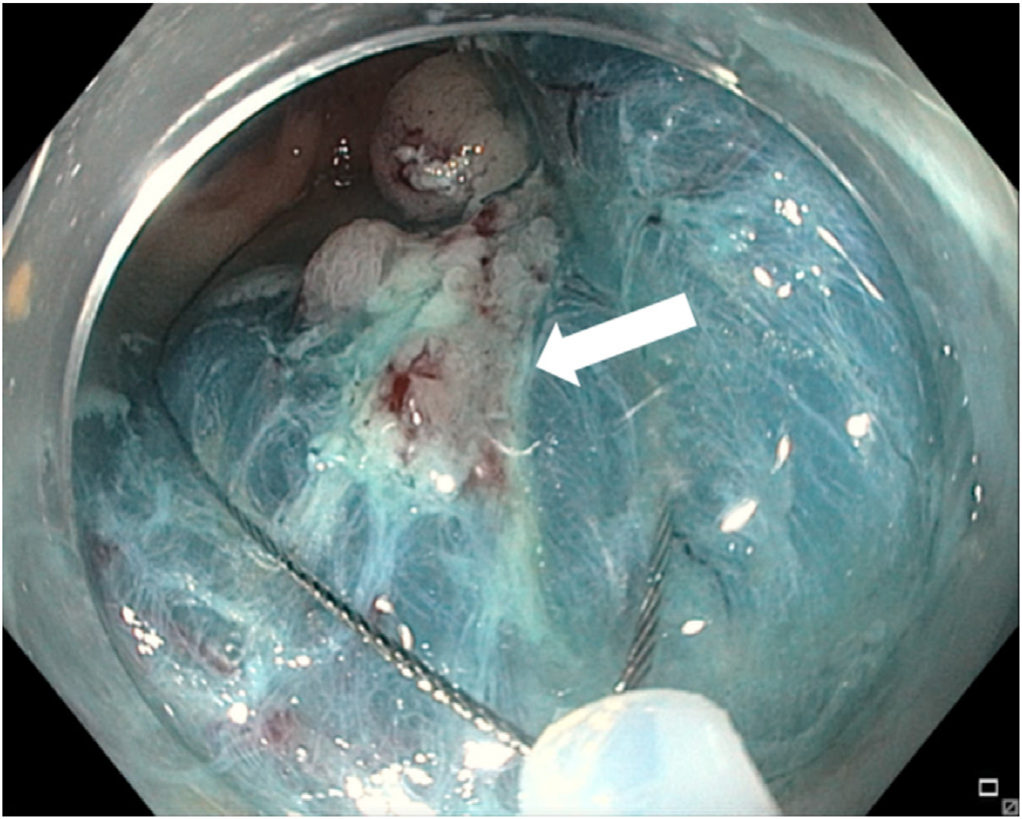
Residual scarred fibrotic tissue at the base of the polyp (*arrow*).

**Figure 7:**
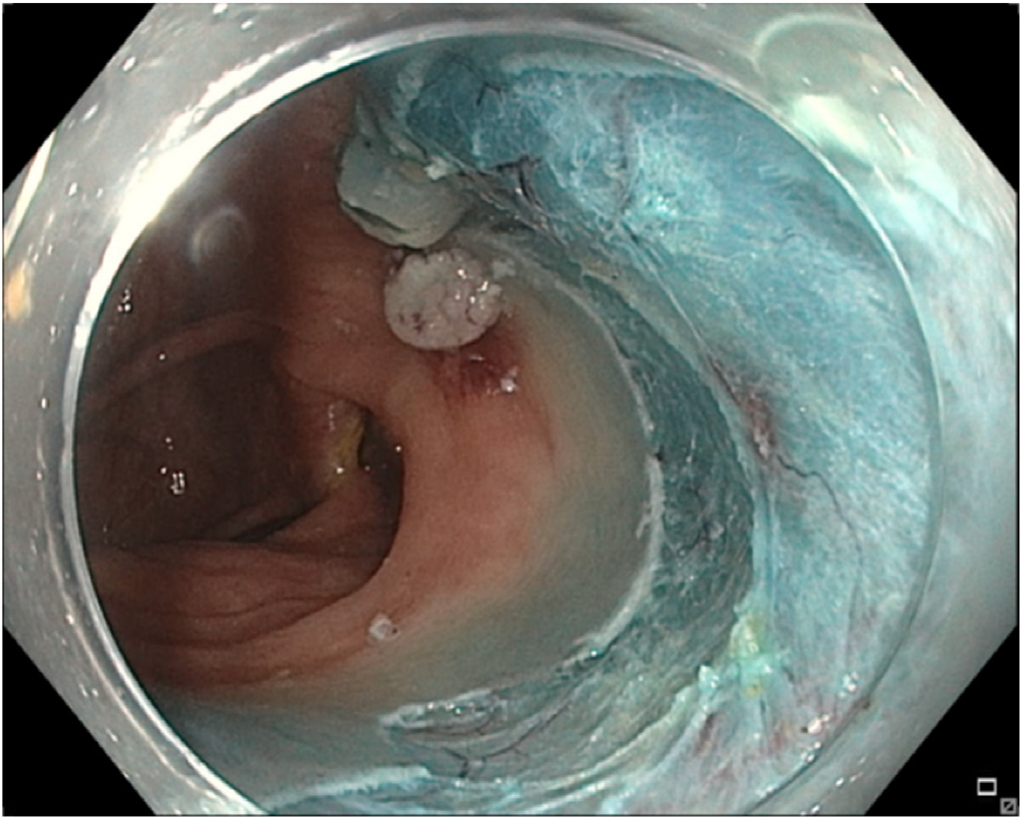
The base of the polyp after resection of fibrotic tissue using the cap-assisted snare capture technique.

A 67-year-old man was referred to our clinic for EMR of a transverse colon polyp. The polyp was of 3-cm diameter, classified as granular laterally spreading tumor (LST), Paris IIa + IIc, and JNET IIb (Fig. 5). A residual fibrotic adenoma tissue was noted after piecemeal EMR of this lesion (Fig. 6). The attempts to capture fibrotic tissue with the snare were unsuccessful because of sliding of the snare over fibrotic tissue. The cap-assisted snare capture technique was used for resection of the remaining fibrotic tissue by using steps described previously. No visible residual adenoma tissue was seen on careful examination of the resection site (Fig. 7). Snare tip soft coagulation of the normal resection margins was performed to decrease the risk of recurrence. The defect was closed with multiple through-the-endoscope clips.

## Case 3

**Figure 8:**
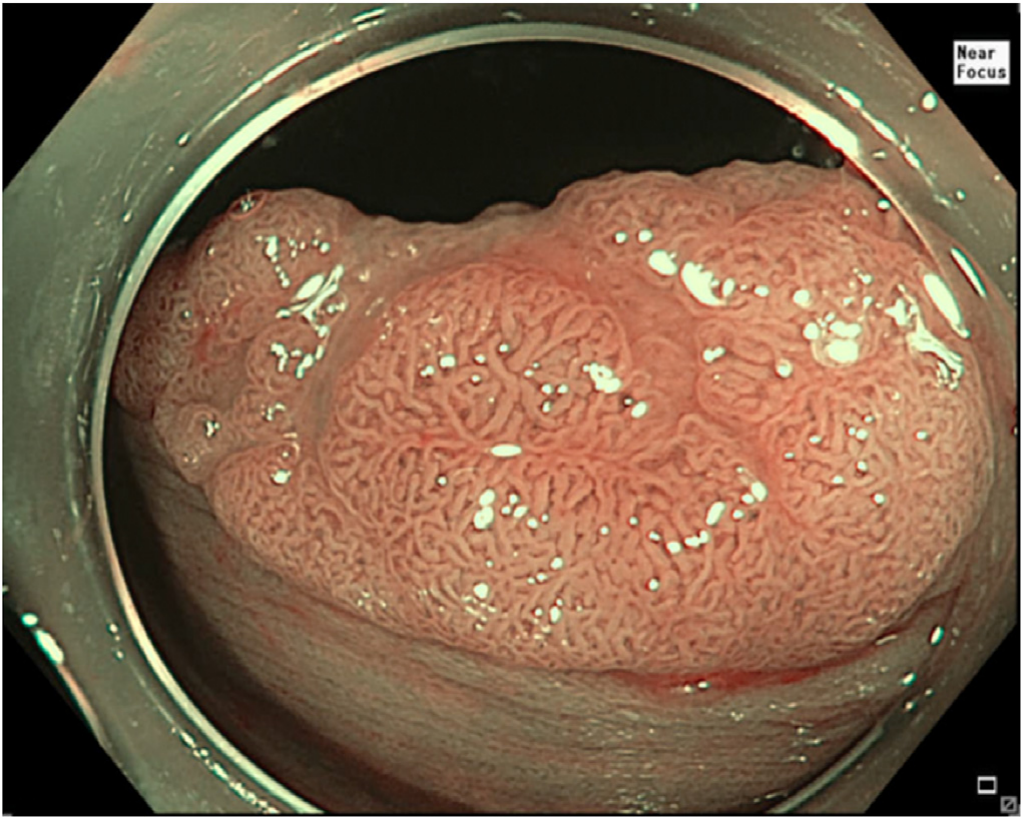
A 3-cm granular, laterally spreading tumor shown in narrow-band imaging view in the descending colon.

**Figure 9:**
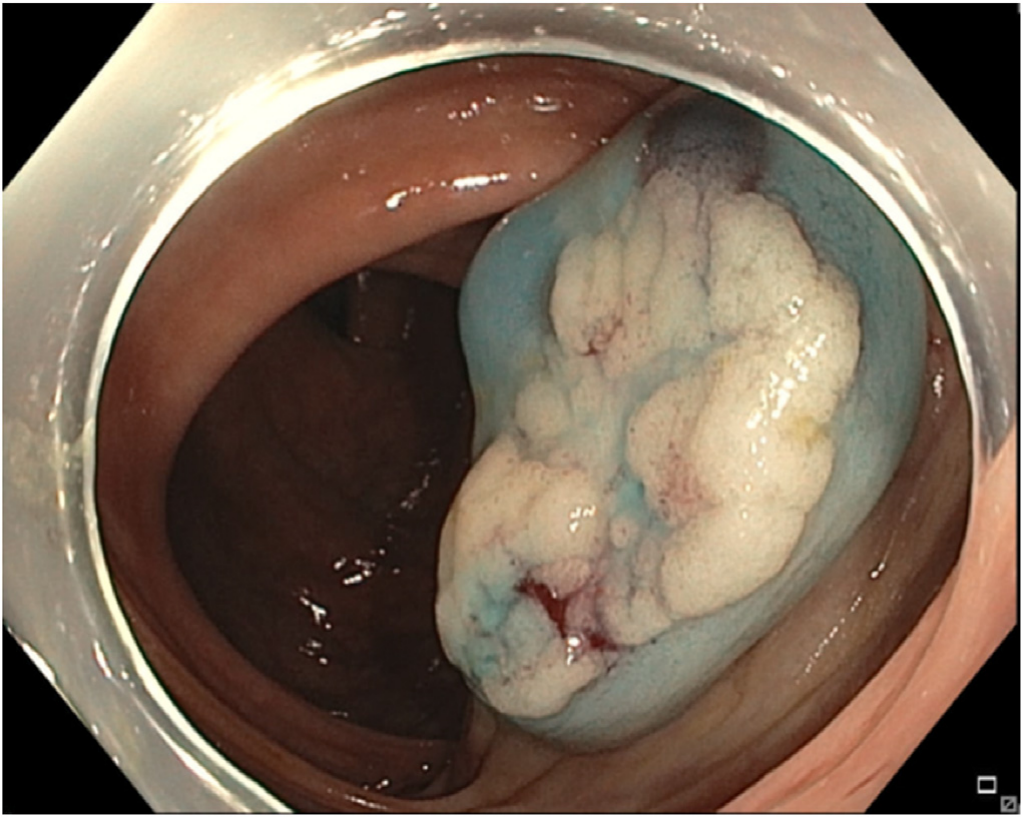
Laterally spreading tumor after lifting with submucosal injection of a starch-based lifting solution.

**Figure 10:**
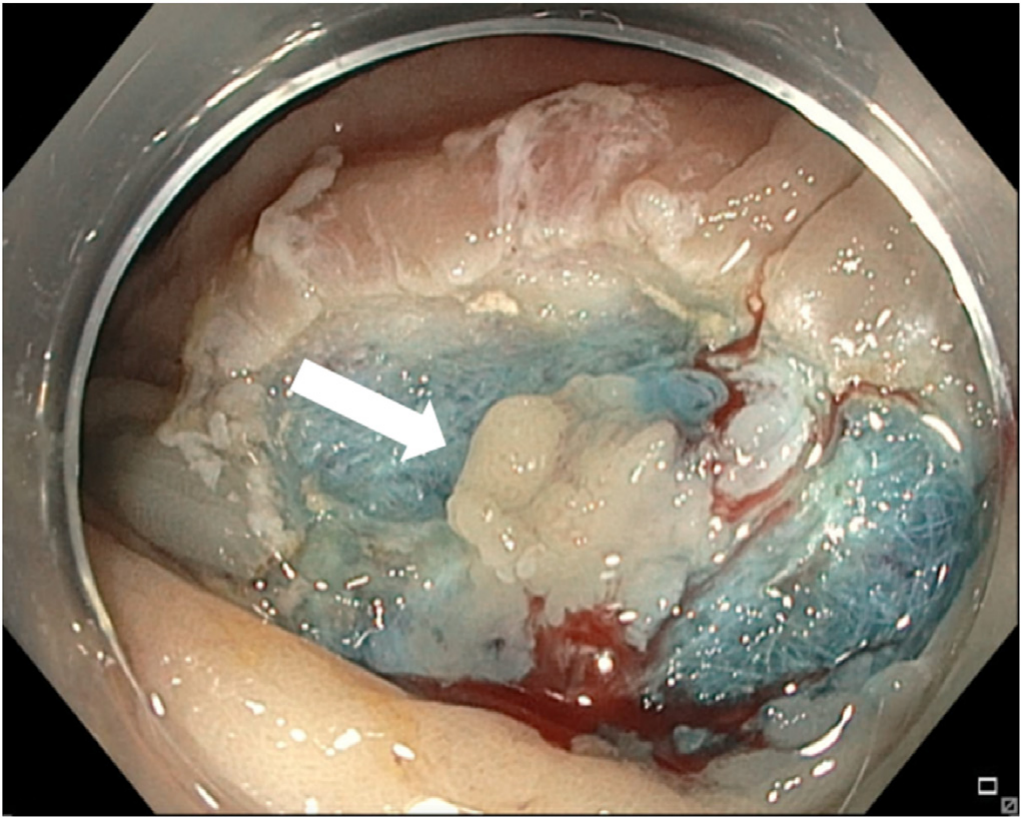
Residual scarred fibrotic tissue at the base of the polyp (*arrow*).

**Figure 11:**
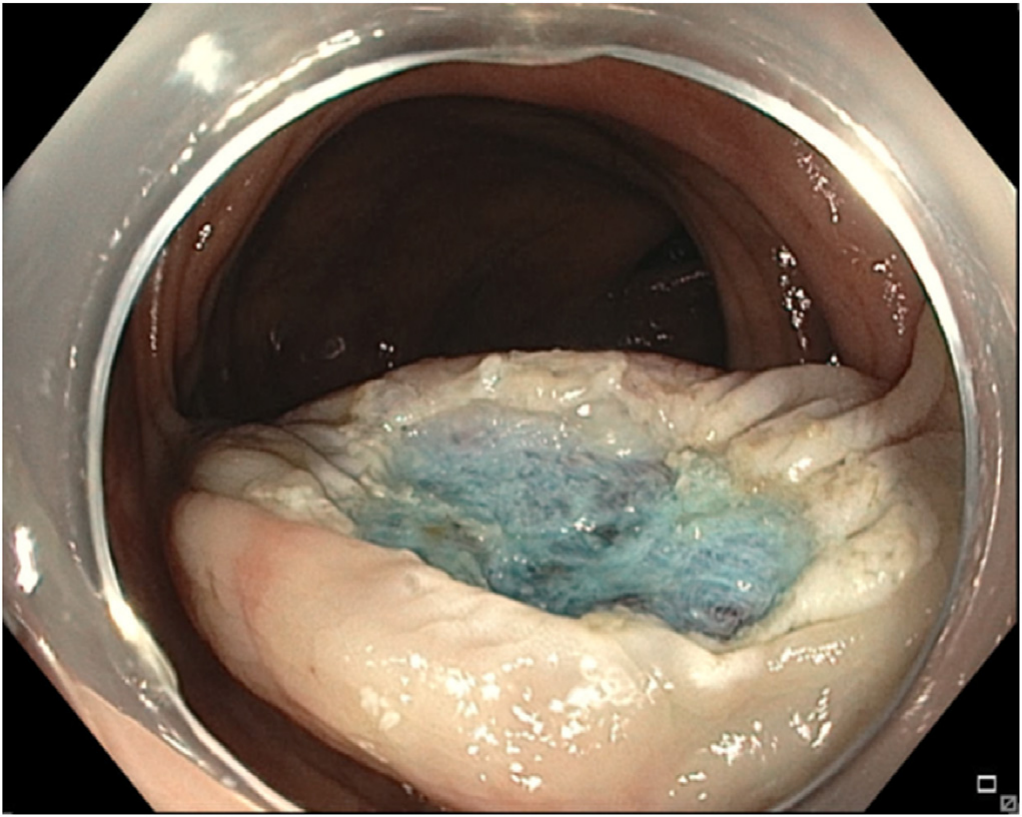
The base of the polyp after resection of fibrotic tissue using the cap-assisted snare capture technique.

A 59-year-old man was referred to our clinic for EMR of a polyp in the descending colon. The polyp was of 3-cm diameter, classified as granular LST, Paris IIa + IIc, and JNET IIb (Figs. 8 and 9). A residual scarred fibrotic tissue was noted after piecemeal EMR of this lesion (Fig. 10). The residual fibrotic tissue was resected using the described steps of a cap-assisted snare capture technique. No visible residual adenoma tissue was seen on careful examination of the resection site (Fig. 11). Snare tip soft coagulation of the normal resection margins was performed to decrease the risk of recurrence. The defect was closed with multiple through-the-endoscope clips.

## Discussion

The cap-assisted snare capture technique is safe and could be an adjunct technique during EMR when conventional snare capture fails. Fibrosis at the site of previous attempts of resection, biopsy, or tattoo injection are common reasons for the failure of conventional snare capture. In general, nonlifting or fibrotic areas are at higher risk for perforation in any endoscopic resection technique. We do not believe the cap-assisted snare capture poses higher risks of perforation than other well-described adjunct techniques such as underwater EMR and hot or cold avulsion. In our experience, the risks of perforation with this technique are low for the following reasons: the use of a small 10- to 15-mm flexible snare captures only the small targeted area under the cap. The distal attachment clear cap extends only a few millimeters, which limits the depth of the snare capture. In our experience, we did not encounter any situation in which the cap blocked the snare closure. In all 3 cases, there was no difficulty in snare closure while maintaining the suction. Placing the snare around the area of interest initially and then maneuvering the endoscope to position the cap over the targeted tissue before applying suction and closing the snare is important. Larger cohort studies are needed to validate the safety and efficacy of this technique.

## Conclusions

Cap-assisted snare capture is a simple technique for resecting fibrotic adenomatous tissue that cannot be captured with conventional snare closure. This technique requires a distal attachment cap and a snare, which are typically used during standard EMR. In our experience, cap-assisted snare capture is a safe and effective adjunct technique when conventional snare capture fails during EMR procedures. This represents a valuable technique in the endoscopists' arsenal for the endoscopic polyp resection.

## Disclosure

Dr Saeed is a consultant for Olympus, Boston Scientific, Medtronic, and EndoGastric Solutions. The other authors have no financial disclosures.

## Supplementary data

Video 1 Cap-assisted snare capture technique for resection of fibrotic large polyps.

